# Effects of Natural Ageing on the Dimensional, Mechanical, and Thermal Properties of Diving Neoprene

**DOI:** 10.3390/polym18202600

**Published:** 2026-10-21

**Authors:** Vesna Marija Potočić Matković, Ivana Salopek Čubrić

**Affiliations:** University of Zagreb Faculty of Textile Technology, 10000 Zagreb, Croatia; ivana.salopek@ttf.unizg.hr

**Keywords:** neoprene, natural ageing, thermal resistance, flexibility, elongation, wetsuit performance

## Abstract

This study examines the effects of natural ageing on thickness, flexibility, elongation, and thermal resistance of neoprene used in wetsuits. A natural ageing protocol was developed to simulate recreational diving conditions under controlled exposure. Thirty-three neoprene specimens representing 11 material types and three thickness categories were repeatedly immersed in seawater at 15 m depth for 45 min per cycle. Fifty immersion, cleaning, and drying cycles were performed, corresponding to 37.5 h of underwater exposure. Thickness decreased in the majority of samples (*p* = 0.00043; Cohen’s d = 0.64), while pre- and post-ageing values remained very strongly correlated (r = 0.9987). Flexibility decreased significantly (*p* = 0.000084; Cohen’s d = −0.74). Thermal resistance declined significantly (*p* = 0.00029; Cohen’s d = 0.66), with greater losses observed in high-performance neoprene. Elongation showed the greatest ageing-related change, decreasing by approximately 25% (*p* < 0.000001; Cohen’s d = 1.32). Thermal resistance was strongly associated with neoprene thickness, whereas thermal-resistance loss was not correlated with thickness reduction (r = −0.085). Overall, natural ageing affected dimensional, mechanical, and thermal properties through multiple degradation processes, highlighting the importance of long-term performance stability in neoprene material selection and wetsuit design.

## 1. Introduction

Neoprene is a cornerstone material in diver thermal protection, combining low thermal conductivity with flexibility and mechanical durability. Previous research has demonstrated a clear relationship between neoprene thickness, thermal resistance, and perceived diver thermal comfort, confirming that increasing neoprene thickness generally improves thermal protection while potentially affecting mobility and comfort under diving conditions [1,2].

While previous work established relationships between neoprene properties and diver thermal comfort, the long-term stability of these properties under realistic diving exposure remains insufficiently understood.

Increasing thickness enhances insulation but can impair mobility and induce user fatigue, especially during prolonged submersion [3].

Neoprene with lower apparent density generally contains a greater proportion of gas-filled cells and therefore provides higher thermal resistance at equivalent thickness, although this advantage may be accompanied by reduced long-term stability. It also restricts movement less and enhances flexibility. Conversely, stiffer, compression-resistant neoprene grades trade off flexibility for durability, making material choice vital for diver performance and comfort. A neoprene suit creates a continuous compression effect during use, meaning that compression is an integral part of the diving suit’s function, not just thermal insulation [4].

In addition to thermal and ergonomic considerations, neoprene must guard against physical hazards such as abrasion and tears—particularly in rugged diving conditions. Lined neoprene variants provide enhanced tensile strength and resistance to mechanical damage.

Numerous accelerated ageing studies have demonstrated that neoprene undergoes significant changes in mechanical performance. Thermal and thermo-oxidative ageing have been associated with increased crosslink density, material stiffening, reduced elongation, and degradation of tensile and tear properties, although the dominant mechanisms depend on exposure conditions and ageing duration [5,6,7,8].

More recently, interest has expanded from purely thermo-oxidative ageing toward degradation in marine environments. Madeira et al. investigated the effects of seawater ageing on carbon black-filled polychloroprene and demonstrated that prolonged seawater exposure alters both the macromolecular network and mechanical properties of the material. Their results showed that seawater absorption initially softened the rubber, while irreversible ageing processes progressively modified the crosslink density and fatigue behavior. The authors emphasized that ageing in marine environments may follow different kinetics and degradation pathways than conventional thermal ageing, highlighting the need for environment-specific durability assessments of polychloroprene-based materials [9].

Despite these advances, the majority of published studies have relied on accelerated thermal, thermo-oxidative, or laboratory-based seawater ageing protocols. Accelerated ageing studies provide valuable mechanistic insight but may not accurately reproduce the combined hydrostatic, thermal, and immersion-related effects experienced by diving neoprene under real service conditions. To the best of our knowledge, few studies have investigated multiple neoprene formulations, thicknesses, and repeated immersion cycles under a unified protocol. This approach offers a controlled and reproducible framework for evaluating the thermal and mechanical degradation of neoprene in diving-relevant environments. While neoprene thickness is widely recognized as the primary determinant of thermal insulation, considerably less is known about how ageing-induced changes in thickness relate to changes in thermal and mechanical performance under realistic diving conditions. Likewise, the role of material density in long-term property retention remains poorly understood.

The aim of this study was to quantify the effects of controlled natural ageing under realistic diving exposure conditions on the dimensional, mechanical, and thermal properties of commercially available neoprene materials differing in thickness and apparent density. Particular attention was given to relationships among ageing-induced changes in thickness, flexibility, elongation, thermal resistance, and density, with the objective of identifying material characteristics associated with long-term performance stability. Unlike our previous studies, which focused on the properties and thermal comfort performance of new, untreated neoprene and on pressure-related changes during diving, the present study investigates changes in the dimensional, mechanical, and thermal properties of neoprene resulting from repeated natural ageing under realistic recreational diving exposure conditions.

## 2. Materials

Neoprene sheets used in this experiment were produced by four well-known manufacturers of quality brands (designation M1, M2, M3, and M4), in eleven different types—no lining (designation NO), one-sided lining (designation OS) and both-sided lining (designation BS), each type in 3 nominal thicknesses (3 mm, 5 mm, and 7 mm), Table 1. These are commonly available thicknesses, but since neoprene sheets are typically manufactured by laminating neoprene with knitted fabrics as lining, real thickness can be slightly higher than nominal. Altogether, 33 different commercially available neoprene sheets were prepared for the analysis to cover a wide interval of the tested characteristics. Commercial names were anonymized to focus the analysis on material behavior rather than brand-specific performance.

Eleven types of neoprene were photographed with a digital microscope at 220× magnification (Table 1). Microscopic images were obtained to provide qualitative insight into structural features. Observed cell diameters ranged approximately from 0.05 mm for smaller cells to 0.2 mm for the largest cells observed, as indicated by the 0.2 mm yellow scale bar on the M1OSa specimen.

Mass per unit area displayed substantial variation, with values between 687.29 g/m^2^ and 1978.56 g/m^2^, averaging 1323.33 g/m^2^. The neoprene sheets examined exhibited a thickness ranging from 3.13 mm to 8.28 mm, with an average of 5.50 mm, reflecting a diverse range of industrially produced neoprene. The apparent density of each neoprene sheet was calculated from the measured thickness and mass per unit area, ranging from 183 kg/m^3^ to 305 kg/m^3^, with an average of 244 kg/m^3^ (Table 2).

## 3. Methodology

### 3.1. Ageing Protocol

Natural ageing of neoprene wetsuits under controlled but realistic diving conditions presents significant logistical challenges. It is practically infeasible to obtain a large sample set of wetsuits that have undergone identical environmental exposure—same water temperature, pressure, duration, and frequency—especially across different neoprene types and thicknesses. To overcome this limitation, 33 newly manufactured neoprene specimens were selected, comprising 11 distinct material types (differing in structure or surface treatment), each in 3 nominal thicknesses (3 mm, 5 mm, and 7 mm). All materials were subjected to a natural ageing protocol designed for this research to simulate the most typical wear conditions during recreational wetsuit use. One independent specimen was used for each combination of material type and nominal thickness, resulting in 33 independent specimens (11 material types × 3 nominal thicknesses). Each specimen was measured before and after ageing, with the number of measurements or replicates for each property determined according to the respective test method.

The exposure protocol was based on dive profiles allowed under the CMAS One Star Diver certification, which permits no-decompression dives to a maximum depth of 20 m. A depth of 15 m was selected as a representative average recreational depth. Each simulated dive lasted 45 min, and 50 immersion cycles were performed, totaling 2250 min (37.5 h) of underwater exposure. Immersion (and cleaning and air-drying) took place between 1 and 15 August in the central Adriatic Sea, where the water temperature at 15 m depth remained constant at 15 °C. Fifty immersion cycles were selected to represent a meaningful level of cumulative diving exposure while remaining experimentally feasible. The objective was not to simulate a specific chronological service life, since wetsuit usage varies considerably among divers, but rather to evaluate the effects of repeated immersion, compression, cleaning, and drying cycles. Such exposure may correspond to several years of occasional recreational use or a substantially shorter period for rental wetsuits subjected to intensive operation.

Following immersion, specimens were rinsed in fresh water, manually cleaned, and then air-dried in the shade to avoid ultraviolet degradation. Drying was performed in the shade to minimize ultraviolet-induced degradation and thereby isolate the effects of seawater immersion, hydrostatic pressure, cyclic compression, and repeated drying. This cycle was repeated 50 times to simulate the drying and handling typical of real-world wetsuit use. The protocol was designed to reproduce not only underwater exposure but also the post-dive handling procedures routinely applied to recreational wetsuits, including rinsing, cleaning, and air drying. Environmental drying conditions ranged from 22 to 33 °C ambient temperature, relative humidity between 32% and 51%, atmospheric pressure between 1013 and 1014 hPa, and wind speeds from 16 to 24 km/h, reflecting a typical Mediterranean summer climate.

In this study, natural ageing refers to repeated exposure under realistic recreational diving and post-dive handling conditions, performed according to a defined protocol; it does not represent the full chronological service life of a wetsuit.

### 3.2. Methods of Measurements

The thickness of neoprene sheets was measured as the vertical distance between the reference plate on which the specimen was placed and the parallel circular plate covering the specimen under pressure, as described in ISO 5084:1996 [10]. A thickness gauge DM-2000 (Wolf, Freiberg, Germany) was used for the test that was conducted as described. During the test, a pressure of 1 kPa was applied to a sample area of 20 cm^2^. A total of 10 measurements were made at different locations on the specimen. The average thickness result was given as the mean value of the 10 measurements.

The mass per unit area of neoprene sheets was measured using the analytic scale Kern ALJ 220-4 (Kern, Balingen-Frommern, Germany) and following the procedure described in international standard ISO 3801:1977 [11].

The apparent density of each neoprene sheet was calculated from the measured thickness and mass per unit area according to:

ρ = mA/t

where ρ is the apparent density (kg m^−3^), mA is the mass per unit area (kg m^−2^), and t is the measured thickness (m). The apparent density was calculated for the complete neoprene sheet as commercially supplied, including any textile lining, and was therefore used as a comparative material descriptor rather than as a measure of the intrinsic density of the neoprene foam.

A sweating guarded hotplate, (Thermetrics, Seattle, WA, USA), a device that simulates the processes of heat and moisture transfer between the human body and environment, was used to measure the thermal resistance of neoprene sheets. The testing was conducted at a temperature of 20 °C, with a relative humidity of 65 ± 1% and an airflow velocity of 1 m s^−1^ according to ISO 11092:2014. The sweating function was not activated because neoprene is considered essentially impermeable to water vapor. The thermal resistance of the neoprene sheet was calculated using the following expression [12]:

(1)  Rct = ((Ts − Ta)/(H/A)) − Rct0

where: Rct—dry thermal resistance of sample only (m^2^ KW^−1^), Ts—hotplate surface temperature (°C), Ta—ambient temperature (°C), H/A—zone heat flux (Wm^−2^), and Rct0—bare plate dry thermal resistance (m^2^ KW^−1^) [12].

Flexibility was determined according to EN 1735:1997 (Method 1—Flat loop method). Rectangular specimens measuring 600 mm × 100 mm were cut in the longitudinal direction of the neoprene sheets. Each specimen was placed on a flat surface and folded to form a loop, while both ends were fixed using a steel plate. After 5 min, the loop height was measured in millimeters. Lower loop height values indicate greater material flexibility [13].

Elongation was determined using a modified strip method based on ISO 13934-1 [14]. Specimens were 50 mm wide, with a gauge length of 100 mm between the clamps, and were cut in the same direction relative to the neoprene sheet. Testing was performed on a Statimat M tensile testing instrument (Textechno, Mönchengladbach, Germany), operating in constant-rate-of-extension mode. The standard test procedure was modified by setting the instrument to stop when a tensile force of 35 N was reached, rather than continuing to the maximum force. Elongation at the applied force of 35 N was recorded as the measure of material extensibility. The same procedure was applied before and after natural ageing. The reference force of 35 N was selected based on technical information from manufacturers of materials for swimwear and adopted as an application-oriented reference for evaluating moderate deformation relevant to wetsuit donning and routine handling, rather than ultimate tensile behaviour.

### 3.3. Statistical Analysis

The normality of paired differences was assessed using the Shapiro–Wilk test. Paired one-tailed *t*-tests were then used to test the hypothesized direction of ageing-induced change for each measured property. Cohen’s d was calculated as a measure of effect size. Statistical analyses were performed using the original unrounded measurement values; rounded values are presented in the tables for clarity. Standard deviations and 95% confidence intervals were calculated across the 33 independent specimens for each property before and after ageing.

## 4. Results and Discussion

Related to the thermal insulation of the investigated neoprene sheets, the results of thermal resistance (Rct) testing suggest a wide spread of insulation levels. The range of thermal resistance for the observed specimens is from 0.036 m^2^ KW^−1^ to 0.206 m^2^ KW^−1^, indicating significantly different overall performance in cold environments. The mean thermal resistance for the observed set equals 0.115 m^2^ KW^−1^. After ageing, the minimum thermal resistance is 0.0354 m^2^ KW^−1^, the maximum thermal resistance is 0.144 m^2^ KW^−1^, and the average is 0.0955 m^2^ KW^−1^, indicating a loss of thermal resistance (Figure 1).

The neoprene sheets exhibited a thickness ranging from 3.13 mm to 8.28 mm, with an average of 5.50 mm. After ageing, the thickness ranged from 3.12 mm to 8.13, with an average of 5.44 mm (Table 3).

The range of bending loop height for the observed specimens is from 3.6 mm to 7.4 mm. The mean loop height for the observed set equals 5.38 mm. After ageing, the minimum loop height is 3.5 mm, the maximum loop height is 7.9 mm, and the average is 5.67 mm. As the measurement standard describes bending the strip into an arc and measuring the height of the resulting arc in mm, a larger arc means less flexibility of the material after aging (Table 3).

The average elongation before aging under a force of 35 N is 130.43%, with a range of 45.3% to 292.2%. After aging, the average elongation under a force of 35 N is 97.67%, with a range of values of 27.3% to 215.40% (Figure 2).

Across the investigated samples, ageing was generally associated with reduced thickness, reduced thermal resistance, reduced elongation, and increased bending loop height, indicating a tendency toward reduced flexibility.

The effects of ageing on the four investigated properties are summarized in Table 4. Thermal resistance and elongation decreased after ageing, whereas increased loop height indicated reduced flexibility, while the change in thickness was comparatively small. The mean values, variability, confidence intervals, and absolute and relative changes are presented for the 33 independent specimens.

### 4.1. Thickness

The present study demonstrates that repeated ageing cycles designed to simulate recreational diving conditions are associated with measurable thickness loss in neoprene materials. Such behaviour is consistent with progressive ageing processes that may affect the structure of neoprene foams through repeated compression, seawater exposure, and recovery cycles [2,9,15].

The normality of the paired thickness differences was not rejected by the Shapiro–Wilk test (*p* > 0.05), indicating that the data are consistent with a normal distribution. A statistically significant difference in sample thickness before and after ageing was identified using a paired *t*-test (*p* = 0.00043), providing strong evidence of a change in thickness following the ageing process (*p* < 0.001). The Pearson correlation coefficient between pre—and post-ageing measurements was exceptionally high (r = 0.9987), indicating that materials that were initially thicker remained relatively thicker after ageing.

The effect size (Cohen’s d = 0.64) corresponds to a medium effect, indicating that the reduction in thickness was not only statistically significant but also practically relevant. Given the established compression behaviour of neoprene under hydrostatic pressure, where thickness decreases with increasing depth [2], the observed ageing-induced thickness reduction suggests that the material’s ability to fully recover its original dimensions may gradually diminish after repeated exposure cycles.

The observed reduction in thickness is consistent with the progressive material changes reported by Madeira et al., who demonstrated that prolonged seawater exposure alters the network structure and mechanical behaviour of polychloroprene rubber. Although their study did not investigate cellular neoprene foams, the reported ageing-induced modifications support the hypothesis that repeated marine exposure may contribute to irreversible structural changes and dimensional instability [9]. Unlike Madeira et al., who investigated bulk polychloroprene rubber under laboratory-controlled seawater ageing, the present study examined dimensional and functional changes in neoprene sheets subjected to repeated recreational diving exposure.

The observed reduction in thickness after the ageing protocol indicates a persistent dimensional change under the conditions investigated. However, the present study does not establish whether this change is irreversible or whether it would be recovered after a longer period of relaxation.

Apparent density showed only weak negative correlations with neoprene thickness before ageing (r = −0.363) and after ageing (r = −0.360), indicating that denser materials tended to be slightly thinner, although the relationship was not strong. Furthermore, density was not associated with ageing-induced thickness loss (r = 0.157), suggesting that dimensional stability is governed by factors other than density alone.

### 4.2. Flexibility

A paired *t*-test comparing flexibility values before and after ageing revealed a highly significant difference (*p* = 0.000084). The Pearson correlation coefficient between pre- and post-ageing flexibility was strong (r = 0.9515), indicating that the relative differences in flexibility between samples were largely preserved after ageing. An effect size of Cohen’s d = −0.74 indicates a medium-to-large effect.

Most samples exhibited increased loop height after ageing, indicating reduced flexibility. The no-lining neoprene samples showed heterogeneous changes in loop height after ageing, with both increases and decreases observed across material types. This variability suggests that the absence of textile reinforcement alone does not determine the ageing response of flexibility.

To evaluate whether changes in flexibility could be predicted from thickness reduction and initial sample characteristics, a multiple linear regression analysis was performed. The model was not statistically significant (R^2^ = 0.13, overall model *p* = 0.25), indicating that the included predictors did not adequately explain variability in flexibility change. Thickness reduction was not significantly associated with flexibility change, while initial thickness showed marginal influence (*p* ≈ 0.098) but remained inconclusive. The poor predictive performance of the model suggests that flexibility changes are governed by factors other than thickness reduction and basic dimensional characteristics.

Although apparent density showed a moderate relationship with the initial flexibility of the investigated neoprenes (r = −0.459), it was not associated with ageing-induced flexibility changes (r = −0.038). Similarly, density was not related to thickness loss during ageing. These findings indicate that flexibility degradation is controlled by factors other than density alone, including foam morphology, textile reinforcement, and material formulation.

However, reduced flexibility and elongation with ageing time were also observed in the study on polychloroprene rubbers by Bouaziz et al. [5].

The reduction in flexibility observed after ageing is consistent with the findings of Madeira et al., who reported significant changes in the mechanical behaviour of polychloroprene following prolonged seawater exposure. Their results suggest that marine ageing can modify the polymer network and fatigue response, leading to altered deformation characteristics and increased material stiffness [9].

The combined molecular and structural alterations were linked to increases in neoprene stiffness and reductions in flexibility in hot air by Xiao et al.’s study [6].

Neither thickness reduction nor apparent density proved to be reliable predictors of flexibility loss during ageing, suggesting that changes in bending behavior arise from more complex structural modifications within the neoprene composite.

### 4.3. Thermal Resistance

A paired *t*-test found a significant reduction in thermal resistance after ageing (*p* = 0.00029). Correlation between pre- and post-ageing values was moderately high (r = 0.7549). The effect size (Cohen’s d = 0.66) indicates a medium effect, meaning the change in thermal resistance is statistically and practically relevant. Three of eleven neoprene groups (M3NOb, M4BS, and M4OS) exhibited higher thermal resistance at comparable thickness than the other investigated materials. This is consistent with the established relationship between neoprene density, cell gas content, and thermal insulation [4]. However, these same samples experienced greater loss in thermal resistance post-ageing, converging with other neoprene in thermal resistance (Figure 1).

Because these three neoprene groups (9 specimens) exhibited distinctly higher thermal-resistance values relative to their thickness, the materials were considered separately in the correlation analysis of thermal resistance. The remaining eight neoprene groups (24 specimens) were also analyzed separately. Within the high-performing group, thickness showed a very strong correlation with initial thermal resistance (r = 0.980), while a similarly strong correlation was observed among the remaining eight groups (r = 0.920). Mass per unit area showed the same pattern, with r = 0.941 for the high-performing group and r = 0.894 for the remaining materials.

After aging, thickness is also highly correlated with thermal resistance for the high-performing group (r = 0.963), and similarly for the remaining eight groups (r = 0.9288). Thickness is the main predictor of thermal resistance, as expected. Although neoprene thickness was strongly correlated with thermal resistance in both the initial and aged states, ageing-induced thickness reduction was not correlated with thermal-resistance loss (r = −0.085). This distinction is important because the initial thermal resistance of the investigated neoprene materials was strongly related to their thickness, whereas the loss of thermal resistance during ageing was not proportional to thickness reduction. The weak correlation between changes in thickness and changes in Rct (r = −0.085) therefore indicates that thickness reduction alone does not explain the observed thermal-resistance loss. This suggests that the factors determining the initial thermal resistance of the investigated materials are not necessarily sufficient to explain the retention of thermal resistance during ageing.

A multiple regression model using thickness reduction, initial thickness, and initial thermal resistance as predictors explained 90.4% of the variance (R^2^ = 0.904; *p* < 0.0001).

The observed reduction in thermal resistance is of practical importance because thermal protection is a primary functional requirement of neoprene garments used by divers, triathletes, swimmers, and rescue personnel. Previous studies have demonstrated strong relationships between neoprene thermal properties, physiological responses, and perceived thermal comfort during aquatic activities [16,17,18,19]. Consequently, even moderate reductions in thermal resistance may influence user comfort and protection during prolonged exposure to cold-water environments [1,20].

### 4.4. Elongation

The average elongation decreased from 130.43% before ageing to 97.67% after ageing, corresponding to an average reduction of approximately 25% (Figure 2). The reduction was highly significant (paired *t*-test, *p* < 0.000001), with a very large effect size (Cohen’s d = 1.32). Among all investigated properties, elongation exhibited the greatest sensitivity to natural ageing.

Elongation exhibited a moderate negative correlation with neoprene thickness before ageing (r = −0.509) and after ageing (r = −0.476), confirming that thinner neoprenes generally showed greater extensibility. A similar relationship was observed between elongation and mass per unit area (r = −0.496), indicating that heavier materials tended to be less extensible. The correlations between thickness and elongation before and after ageing were calculated across all 33 specimens pooled together and therefore represent associations within the investigated dataset rather than universal material relationships. The observed correlations may also reflect differences among the investigated commercial materials, including their formulation and construction. In contrast, elongation showed no meaningful relationship with apparent density (r = 0.138), suggesting that stretch behaviour is governed primarily by material construction, textile reinforcement, and neoprene formulation rather than foam density.

The correlation between elongation before and after ageing was very high (r = 0.972), indicating that materials that were initially more extensible remained relatively more extensible after ageing. However, the magnitude of elongation loss was not significantly associated with thickness reduction (r = −0.2454), loss of flexibility (r = 0.230), or thermal-resistance loss (r = 0.2810). Interestingly, elongation loss showed a weak-to-moderate negative correlation with initial apparent density (r = −0.363, *p* = 0.038), suggesting that denser materials may experience somewhat greater losses in extensibility during ageing, although density was not associated with initial elongation.

The observed reduction in elongation is consistent with the findings of Bouaziz et al., who reported ageing-induced changes in the mechanical response of polychloroprene associated with modifications of the polymer network. Similar ageing-related increases in stiffness and reductions in deformability have also been reported for neoprene exposed to elevated temperatures and oxidative conditions. The present results demonstrate that comparable effects occur under realistic diving exposure conditions involving repeated seawater immersion, hydrostatic compression, and drying cycles [5,6].

### 4.5. Integrated Ageing Effects on Neoprene Performance

The ageing-induced changes observed in the investigated neoprene materials appeared largely independent of one another. Reductions in thickness, elongation, flexibility, and thermal resistance showed only weak mutual correlations, suggesting that natural ageing does not affect all performance characteristics through a single dominant degradation mechanism. The different response patterns suggest that the changes in the investigated properties cannot be explained by a single measured parameter.

Particularly noteworthy was the absence of a relationship between thickness reduction and thermal-resistance loss (r = −0.085). Although thickness was strongly associated with thermal resistance in both the initial and aged states, changes in thermal performance could not be explained solely by dimensional changes. This finding suggests that ageing-related changes in material characteristics may contribute significantly to insulation degradation.

Apparent density showed little influence on dimensional stability, flexibility, or elongation but exhibited a moderate association with thermal-resistance loss (r = 0.522). These results suggest that material characteristics related to apparent density may play a more important role in long-term thermal stability than in the evolution of mechanical properties.

## 5. Conclusions

Natural ageing under controlled diving exposure conditions induced statistically and practically significant changes in all investigated neoprene properties.

Thickness decreased overall across the investigated samples (*p* = 0.00043; Cohen’s d = 0.64), indicating measurable dimensional degradation. However, thickness loss was not associated with changes in thermal resistance or elongation, suggesting that dimensional changes alone cannot explain ageing-related performance loss.

Flexibility was significantly affected by ageing (*p* = 0.000084; Cohen’s d = −0.74). The flexibility response varied among unlined and lined materials, indicating that textile reinforcement alone does not determine ageing behaviour. Changes in flexibility could not be reliably predicted from thickness-related variables or apparent density.

Thermal resistance declined significantly after ageing (*p* = 0.00029; Cohen’s d = 0.66). High-performance neoprene exhibited higher initial thermal performance but also greater thermal-resistance loss. The weak association between thickness reduction and thermal-resistance loss indicates that dimensional change alone does not account for the reduction in thermal resistance during ageing.

Elongation was the property most strongly affected by ageing, decreasing by approximately 25% on average (*p* < 0.000001; Cohen’s d = 1.32). Despite substantial reductions in extensibility, materials that initially exhibited greater elongation generally remained relatively more extensible after ageing. Initial apparent density showed a weak-to-moderate negative correlation with elongation loss (r = −0.363, *p* = 0.038), suggesting that denser materials tended to exhibit greater reductions in elongation.

Overall, the results indicate that natural ageing affects thermal, dimensional, and mechanical properties to different extents, and that changes in one property cannot necessarily be used to predict changes in another. The results further suggest that neoprene formulations offering superior initial thermal insulation may be more susceptible to long-term thermal-performance loss under repeated diving exposure.

These findings highlight the importance of incorporating ageing behavior into neoprene material selection and design, particularly for applications requiring sustained thermal protection and mechanical performance throughout the service life of the garment.

## Figures and Tables

**Figure 1:**
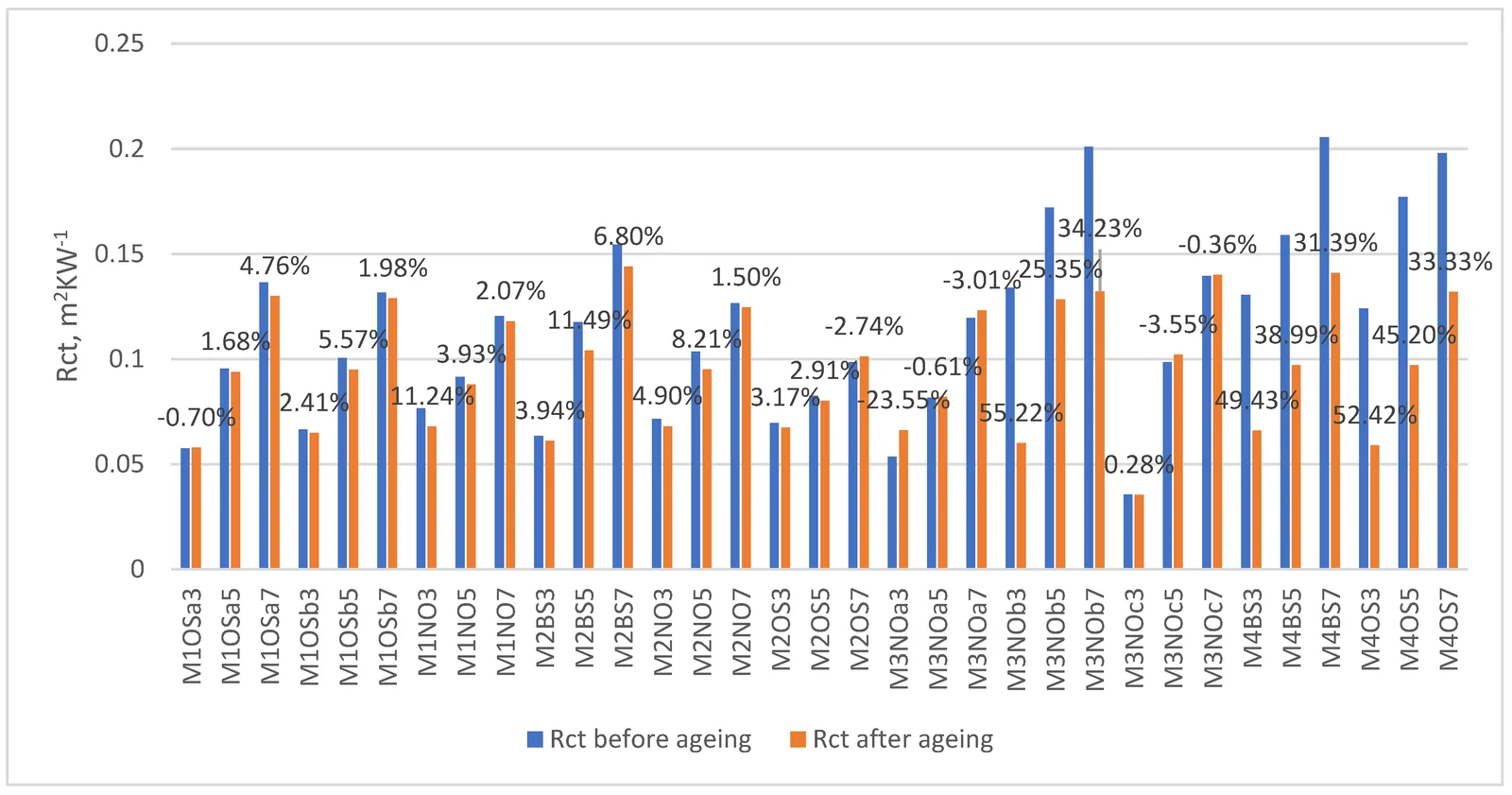
Thermal resistance (Rct) of individual neoprene specimens before and after ageing, with percentage change after ageing (positive values indicate a reduction in Rct, whereas negative values indicate an increase).

**Figure 2:**
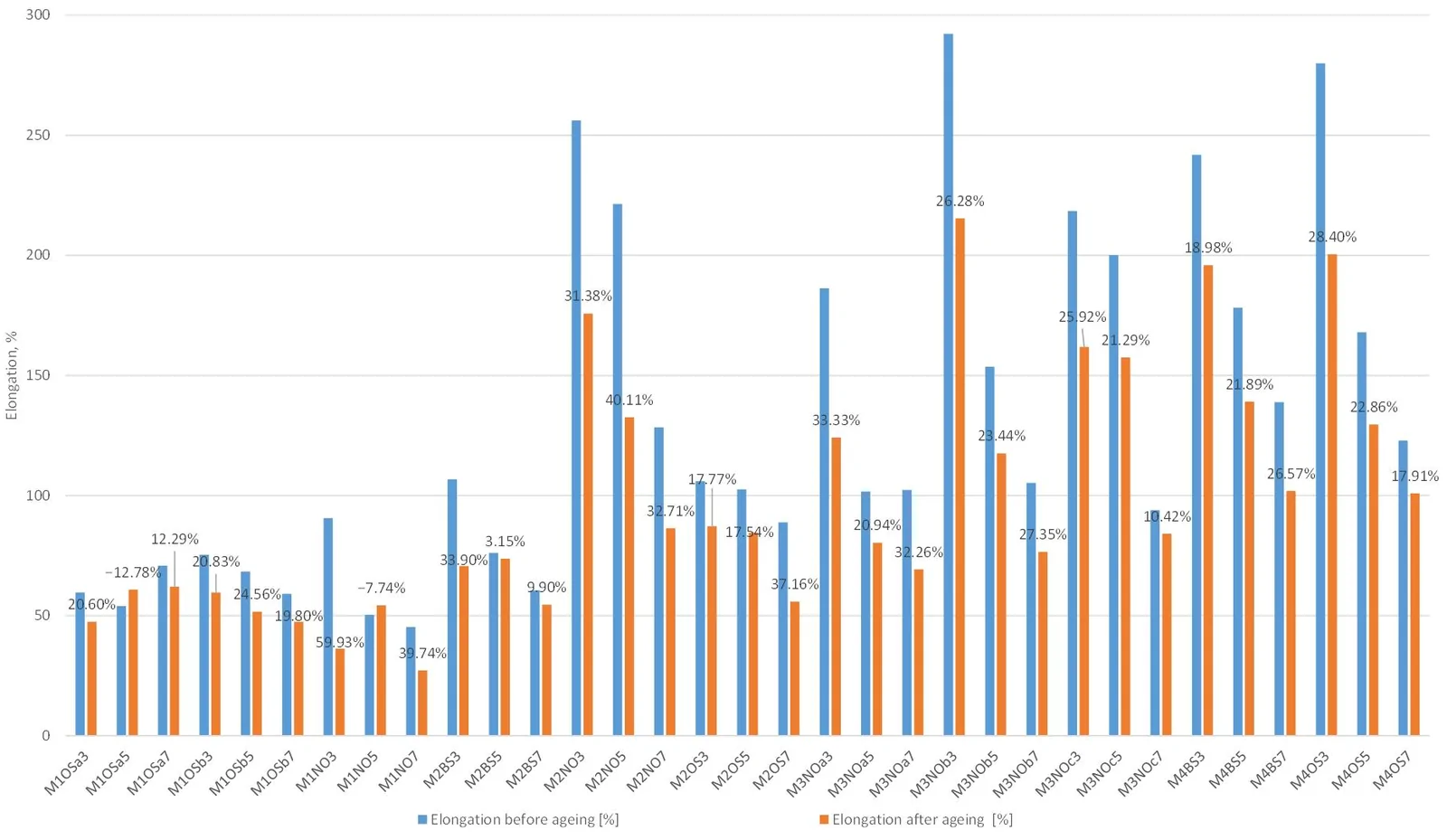
Elongation of individual neoprene specimens before and after ageing, with percentage change after ageing (positive values indicate a reduction in elongation, whereas negative values indicate an increase).

**Table 1:** Digital microscope photograph at 220× magnification of 11 types of neoprene.

M1OSa	M1OSb	M1NO
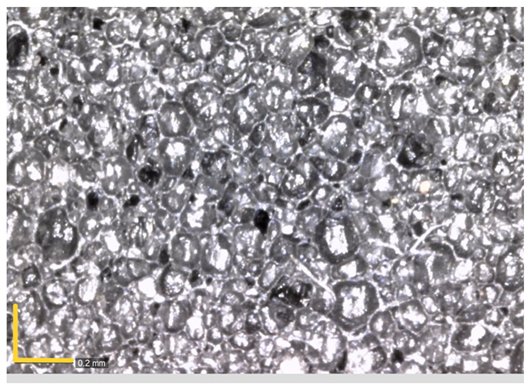	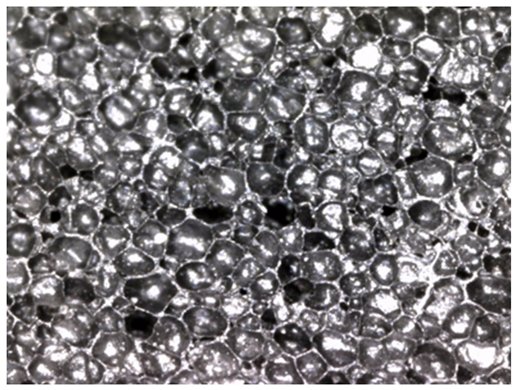	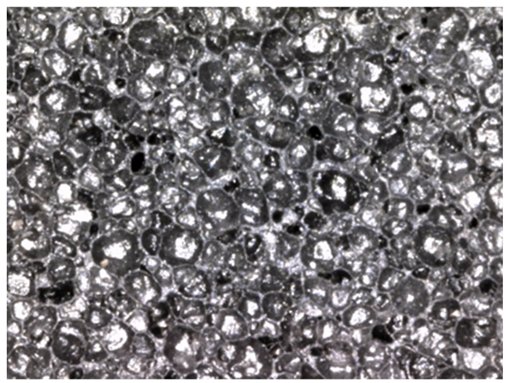
M2BS	M2NO	M2OS
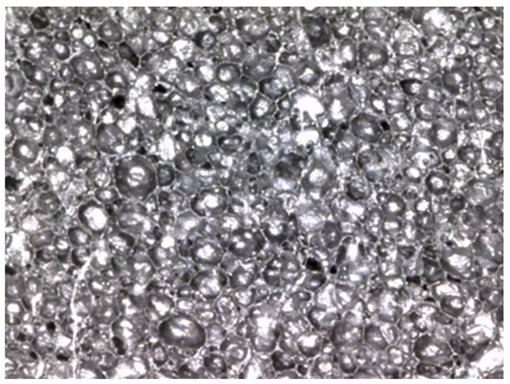	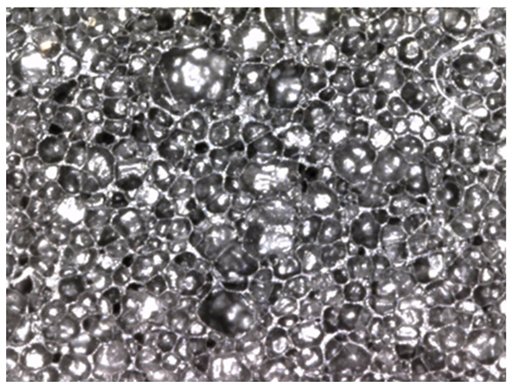	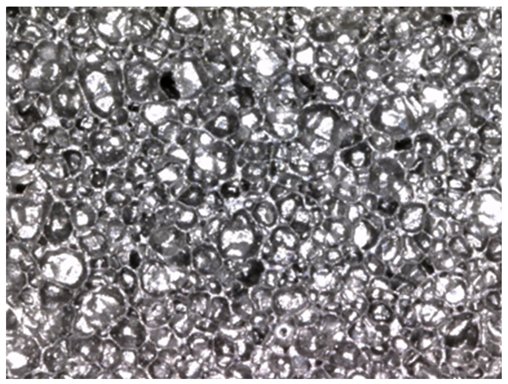
M3NOa	M3NOb	M3NOc
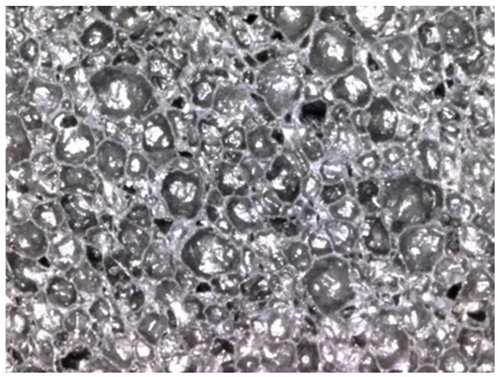	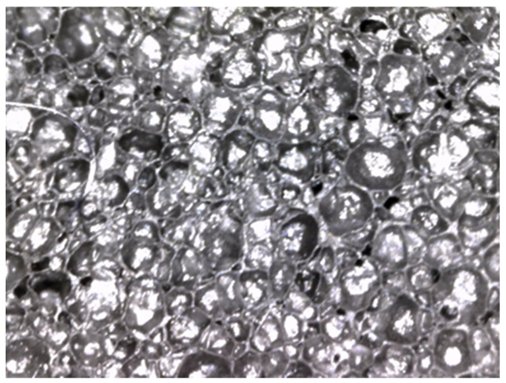	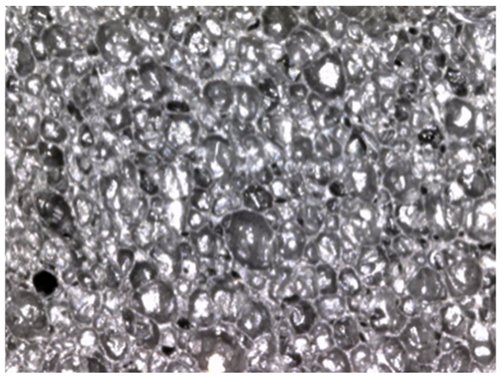
M4BS	M4OS	
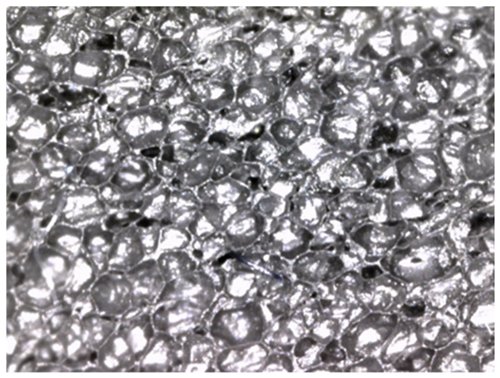	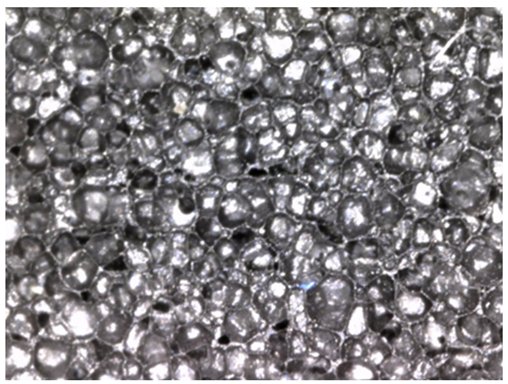	

**Table 2:** The basic characteristics of neoprene sheets.

Sample	Thickness (mm)	Mass per Unit Area (g/m^2^)	Apparent Density (kg/m^3^)
M1OSa3	3.59	826.61	230
M1OSa5	5.76	1380.69	240
M1OSa7	7.62	1668.22	219
M1OSb3	3.74	945.70	253
M1OSb5	5.50	1277.17	232
M1OSb7	7.40	1835.69	248
M1NO3	3.77	1104.54	293
M1NO5	5.15	1283.13	249
M1NO7	6.94	1755.99	253
M2BS3	3.81	968.12	254
M2BS5	6.98	1277.00	183
M2BS7	8.00	1966.54	246
M2NO3	3.76	1148.23	305
M2NO5	5.10	1458.62	286
M2NO7	7.22	1808.25	251
M2OS3	3.67	895.70	244
M2OS5	5.24	1277.00	244
M2OS7	8.28	1978.56	239
M3NOa3	3.29	874.61	266
M3NOa5	4.66	1382.11	297
M3NOa7	6.74	1820.76	270
M3NOb3	3.45	988.29	286
M3NOb5	5.52	1288.65	234
M3NOb7	7.07	1659.18	235
M3NOc3	3.13	858.12	274
M3NOc5	5.25	1323.01	252
M3NOc7	7.63	1933.70	254
M4BS3	3.70	759.59	205
M4BS5	5.72	1202.27	210
M4BS7	7.74	1538.36	199
M4OS3	3.32	687.29	207
M4OS5	5.57	1093.46	196
M4OS7	7.20	1404.61	195

**Table 3:** Thickness and flexibility before and after ageing of neoprene sheets.

Sample	Thickness (mm)	Thickness Aged (mm)	Flexibility (mm)	Flexibility Aged (mm)
M1OSa3	3.590	3.570	3.9	4.3
M1OSa5	5.757	5.490	5.1	5.8
M1OSa7	7.623	7.523	6.4	7.0
M1OSb3	3.740	3.737	4.0	4.7
M1OSb5	5.503	5.497	5.8	6.6
M1OSb7	7.397	7.330	6.7	7.4
M1NO3	3.767	3.617	4.3	4.2
M1NO5	5.147	5.063	5.1	5.1
M1NO7	6.940	6.843	6.6	6.3
M2BS3	3.807	3.743	4.4	4.6
M2BS5	6.983	6.797	6.0	7.0
M2BS7	8.000	8.053	7.2	7.6
M2NO3	3.763	3.667	4.4	4.6
M2NO5	5.100	5.043	5.1	6.0
M2NO7	7.217	7.177	6.4	7.4
M2OS3	3.673	3.643	4.0	4.0
M2OS5	5.237	5.293	5.7	5.9
M2OS7	8.283	8.133	7.4	7.9
M3NOa3	3.293	3.283	3.7	3.5
M3NOa5	4.657	4.677	4.6	5.0
M3NOa7	6.743	6.741	6.1	6.0
M3NOb3	3.450	3.470	3.7	3.7
M3NOb5	5.517	5.627	5.2	5.4
M3NOb7	7.067	7.030	6.6	6.3
M3NOc3	3.127	3.120	3.6	3.6
M3NOc5	5.247	5.247	5.1	5.0
M3NOc7	7.627	7.437	6.3	7.0
M4BS3	3.697	3.697	5.2	5.6
M4BS5	5.717	5.467	5.8	5.9
M4BS7	7.737	7.697	6.8	6.8
M4OS3	3.317	3.330	4.3	4.4
M4OS5	5.573	5.557	5.9	6.0
M4OS7	7.197	7.070	7.1	6.7

**Table 4:** Mean values, standard deviations, 95% confidence intervals, and changes in the measured properties before and after ageing.

Property	Before Ageing, Mean ± SD	95% CI	After Ageing, Mean ± SD	95% CI	Absolute Change	Change (%)
Thermal resistance, Rct (m^2^ K W^−1^)	0.1149 ± 0.0444	0.0992–0.1307	0.0955 ± 0.0301	0.0848–0.1062	−0.0195	−16.93
Thickness (mm)	5.4997 ± 1.6646	4.9095–6.0900	5.4445 ± 1.6408	4.8627–6.0263	−0.0552	−1.00
Flexibility (mm)	5.3845 ± 1.1430	4.9793–5.7898	5.6722 ± 1.2525	5.2281–6.1164	+0.2877	+5.34
Elongation (%)	130.4294 ± 71.0961	105.2198–155.6390	97.6709 ± 50.5725	79.7387–115.6031	−32.7585	−25.12

SD and 95% CI describe variability among the 33 independent specimens.

## Data Availability

The original contributions presented in this study are included in the article. Further inquiries can be directed to the corresponding author.
